# A Lexicon of DNA Modifications: Their Roles in Embryo Development and the Germline

**DOI:** 10.3389/fcell.2018.00024

**Published:** 2018-03-27

**Authors:** Qifan Zhu, Reinhard Stöger, Ramiro Alberio

**Affiliations:** School of Biosciences, University of Nottingham, Nottingham, United Kingdom

**Keywords:** modified bases in eukaryotic DNA, 5mC, 5hmC, 6mA, germ cells, embryo, epigenetic reprogramming

## Abstract

5-methylcytosine (5mC) on CpG dinucleotides has been viewed as the major epigenetic modification in eukaryotes for a long time. Apart from 5mC, additional DNA modifications have been discovered in eukaryotic genomes. Many of these modifications are thought to be solely associated with DNA damage. However, growing evidence indicates that some base modifications, namely 5-hydroxymethylcytosine (5hmC), 5-formylcytosine (5fC), 5-carboxylcytosine (5caC), and N6-methadenine (6mA), may be of biological relevance, particularly during early stages of embryo development. Although abundance of these DNA modifications in eukaryotic genomes can be low, there are suggestions that they cooperate with other epigenetic markers to affect DNA-protein interactions, gene expression, defense of genome stability and epigenetic inheritance. Little is still known about their distribution in different tissues and their functions during key stages of the animal lifecycle. This review discusses current knowledge and future perspectives of these novel DNA modifications in the mammalian genome with a focus on their dynamic distribution during early embryonic development and their potential function in epigenetic inheritance through the germ line.

## Introduction

5-methylcytosine (5mC) in CpG dinucleotides is the most abundantly modified DNA base in eukaryotes. Its role as an epigenetic regulator of gene expression has been widely studied and documented (Holliday and Pugh, 1975; Riggs, 1975; Sager and Kitchin, 1975; Bird and Wolffe, 1999; Bird, 2002; Robertson, 2005; Jones, 2012; Smith and Meissner, 2013; Edwards et al., 2017). However, DNA modifications are much more diverse than previously thought. Apart from 5mC, nearly 40 verified and many more unverified DNA modifications can be found in a newly established DNA modification Database (Sood et al., 2016). Novel approaches have been suggested for systematic detection (Thiaville et al., 2016) and computational identification (Iyer et al., 2013) of novel epigenetic marks in prokaryotes, of which the strategies could also be applied to eukaryotic genomes. Most of the DNA modifications are indicators of DNA damage and related to DNA repair pathways (Ito and Kuraoka, 2015), but they have not yet been correlated with other biological processes. Studies in the last few years provide growing evidence for biological significance of some DNA modifications, including 5-hydroxymethylcytosine (5hmC), 5-formylcytosine (5fC), 5-carboxylcytosine (5caC), 5-hydroxymethyluracil (5hmU), and N6-methadenine (6mA). With recently suggested biological potentials, these DNA base variants are considered as ‘novel DNA modifications’. The rareness and instability of these novel DNA modifications in eukaryotic genomes are the major obstacles for studying their potential functions. Because of these features, most of the aforementioned novel modifications were considered to be intermediates of DNA demethylation processes, and doubts have been cast on whether they are abundant or sufficiently stable to have significant effects on biological processes. Whether and how these novel DNA modifications can be stably maintained through cell division and during differentiation is largely unknown. Still, these modifications have been suggested to cooperate with histone modifications and thereby affect the epigenome at specific loci. They also affect the structure and accessibility of DNA, which in turn regulates DNA-protein interactions, gene expression and genome stability. Although little is known about their distribution, their turnover and their functions, these novel DNA modifications show highly temporal and spatially restricted profiles. For example, 6mA shows significant but transient enrichment in early embryogenesis of zebrafish and pig, with a maximum of ~0.1% 6mA/A in 32-cell to 64-cell embryo stage in zebrafish and ~0.17% in pig 4-cell to morula stage, but only 0.006% in zebrafish 512-cell stage and 0.05% in pig blastocyst (Liu J. et al., 2016). Distributions and regulatory mechanisms of DNA modifications also differ greatly among species (Law and Jacobsen, 2010; He Y.-F. et al., 2011). For example, compared to mammalian genomes, a higher proportion (over 30%) of 5mC in plant genomes is located within non-CG sites (Vanyushin and Ashapkin, 2011), whereas *C. elegans* and *D. melanogaster* genomes are largely devoid of 5mC (Capuano et al., 2014). Similar to other well-studied epigenetic marks, novel DNA modifications are highly dynamic during the two waves of global epigenetic reprogramming at early embryonic stages in mammals. However we still know very little about their roles during these events. This review will discuss the current knowledge and future perspectives of these novel DNA modifications as well as 5mC, with a focus on their profiles and potential biological function in early embryonic development and the germline in mammals.

## 5mC and tet-mediated active demethylation in early development

The presence of 5-methylcytosine in DNA (5mC) was first reported in mammals in the late 1940s and its role in epigenetic regulation of gene expression has been widely studied and documented (Jones, 2012; Plongthongkum et al., 2014; Breiling and Lyko, 2015). The functions of 5mC in early development are mainly revealed via studies of 5mC distribution and activities of its modifiers in embryonic stem cells (ESCs). 5mC in promoter regions—upstream of transcription start sites (TSS)—and transposable elements, is generally considered as a silencing mark for gene expression, while the regulatory role of 5mC on gene bodies for gene expression remains unclear (Jones, 2012; Kim et al., 2014; Chen et al., 2016; Hargan-Calvopina et al., 2016). DNA methylation at distal regulatory elements, such as embryonic stem cell (ES)-specific enhancers and insulators, has also been suggested to correlate with gene expression (Hon et al., 2013; Aran et al., 2016; Heyn et al., 2016) as well as with the activities of transcriptional regulators (Stadler et al., 2011; Wang et al., 2012; Maurano et al., 2015; Aran et al., 2016; Hnisz et al., 2016). For example, triple-knockout (TKO) of *Tet* (Ten-eleven translocation) enzymes, the 5mC ‘editors’, in mouse ESCs (mESCs) results in increased DNA methylation mainly at distal enhancer regions coupled with changed expression of linked genes, suggesting that Tet-dependent active demethylation could participate in the regulation of distal regulatory regions (Hon et al., 2014; Lu et al., 2014).

In mammals, two waves of epigenetic reprogramming featuring global DNA demethylation take place in preimplantation embryos and primordial germ cells (PGCs). This genome-wide DNA demethylation involves inhibition of the 5mC-maintenance DNA methyltransferase 1 (Dnmt1)/Ubiquitin-Like with PHD and Ring Finger Domains 1 (Uhrf1) enzyme complex and ‘*de novo*’ DNA methyltransferases 3a/b (Dnmt3a/b), followed by dilution of 5mC during DNA replication; a process also known as passive demethylation. Active, enzyme-mediated ‘methyl-group’ removal mechanism(s), in which 5mC is oxidized sequentially via Tet proteins into 5-hydroxymethylcytosine (5hmC), 5-formylcytosine (5fC) and 5-carboxylcytosine (5caC), are also critical for epigenetic reprogramming.

### Global demethylation in preimplantation embryo

In mice, soon after fertilization, male and female pronuclei show differential demethylation kinetics (Mayer et al., 2000; Oswald et al., 2000). These observations have led to a model for DNA demethylation in which the male pronucleus undergoes active demethylation mediated by Tet3 (Iqbal et al., 2011). 5hmC and other oxidative products generated by TET proteins are removed via the base excision repair (BER) pathway as well as replication-dependent dilution (Gu et al., 2011; Inoue and Zhang, 2011; Iqbal et al., 2011). In contrast slower demethylation kinetics are observed in the maternal pronucleus where histone H3Lys9-dimethylation marked chromatin (H3K9me2) has a tighter association with protein Stella/DPPA3 compared to the paternal pronucleus. This chromatin state has been suggested to protect the maternal pronucleus from active, Tet3-mediated demethylation (Nakamura et al., 2007, 2012; Messerschmidt, 2012). The finding that Tet3 predominantly localizes on paternal pronuclei further supports this model (Gu et al., 2011; Nakamura et al., 2012). Bisulfite sequencing and mass spectrometry have also shown pronounced demethylation in the zygote with different demethylation kinetics between paternal and maternal pronuclei (Guo F. et al., 2014; Okamoto et al., 2016). However, investigations at single-base-resolution show that Tet-mediated demethylation is operational in the maternal genome, albeit with slower kinetics compared to the paternal genome (Guo F. et al., 2014; Guo H. et al., 2014; Tsukada et al., 2015; Zhu et al., 2017).

While global demethylation persists until the late blastocyst stage (Okamoto et al., 2016), *Tet3* expression rapidly diminishes at the 2-cell stage (maternal-zygotic transition) and is replaced by *Tet1/2* around the morula stage (Gu et al., 2011; Iqbal et al., 2011; Cao et al., 2014; Lee K. et al., 2014; Gao et al., 2017). Genome-wide methylation is restored in late blastocysts and continues during germ layer establishment (Inoue and Zhang, 2011; Guenatri et al., 2013). During this developmental stage, Dnmt3a/b exhibit distinct sequence preferences and target diverse genomic regions (Okano et al., 1999; Watanabe et al., 2002; Chen et al., 2004; Borgel et al., 2010; Velasco et al., 2010; Auclair et al., 2014; Liao et al., 2015), while Dnmt3l acts as cofactor (Ooi et al., 2007; Jurkowska et al., 2011). Overall, remodeling of the embryonic methylome has been suggested to be a double-check security system, in which the global demethylation contributes to the establishment of totipotency and prevents any potentially deleterious epigenetic inheritance, while the re-establishment of methylation is crucial for exit of totipotency/pluripotency and for efficient spatiotemporal control of genome activity and lineage decision (Guenatri et al., 2013; Lee H. J. et al., 2014).

A recent study suggested that in the mouse paternal genome Tet3 may function as an antagonist to *de novo* methylation activity in later zygotic stages, but is not necessarily required for demethylation before pronuclear stage 3 (PN3), as Tet3-independent 5mC erasure occurs before 5hmC is generated (Amouroux et al., 2016). Although Tet3-mediated active demethylation has been linked with paternal genome activation and activities of pluripotent genes such as *Oct4* and *Nanog* (Ito et al., 2010; Guo F. et al., 2014), deletion of maternal Tet3 (Gu et al., 2011) does not seem to affect pre-implantation development. This suggests that Tet3 functions may be compensated by the other two Tet enzymes, or defects in DNA active demethylation can be tolerated during pre-implantation development. Heterozygous Tet3-KO mice show neonatal sub-lethality, suggesting that Tet3 disruption at early embryonic stages may affect later development (Gu et al., 2011; Inoue et al., 2015; Tsukada et al., 2015). It is possible that passive demethylation on the paternal genome is sufficient for preimplantation development. That is, partial impairment of symmetric maintenance DNA methylation at CpG dyads, rather than active demethylation, is thought to be the main driver of DNA demethylation of both paternal and maternal genome at the zygote stage and also in PGCs (Guo H. et al., 2014; Arand et al., 2015).

DNA methylation dynamics have also been studied in species other than mouse. Although the demethylation profiles in human and mice are generally similar (Smith et al., 2014), differences have been observed between the 2-cell to morula stages, such as an increase of methylation level at pronuclear stage and 4- to 8-cell stages in human embryo (Guo H. et al., 2014; Zhu et al., 2018). In humans rapid demethylation and hydroxymethylation are also observed in both male and female pronuclei, suggesting TET-mediated active demethylation on both the paternal and maternal genome (Guo H. et al., 2014). Similar to mouse (Salvaing et al., 2012; Li et al., 2016), 5mC and 5hmC co-exist in both pronuclei of human zygotes and in oocytes in a non-reciprocal pattern (Petrussa et al., 2016), which is consistent with the detection of *de novo* methylation at PN3 (Amouroux et al., 2016). In Rhesus monkey, a methylation peak was also reported at 8-cell stage, and *de novo* methylation was observed at some paternal and maternal CpG sites at 2-cell stage and onwards. Single-cell DNA methylome data of preimplantation human embryo further indicates *de novo* methylation is enriched for major families of repetitive elements, especially evolutionarily younger subfamilies, such as ALU and LINE1 retroelements (Zhu et al., 2018). These data indicate that, unlike the generally unidirectional demethylation suggested before, *de novo* methylation could be concurrent with and even ‘outpace’ DNA demethylation in preimplantation stages (Gao et al., 2017). It will be interesting to elucidate the mechanisms that support these transient waves of DNA methylation and their importance for normal development.

DNA methylation and hydroxymethylation profiling in early embryos of domestic animals, including bovine (Dobbs et al., 2013), porcine (Cao et al., 2014; Lee K. et al., 2014), ovine (Jafarpour et al., 2017; Masala et al., 2017), and equine (Heras et al., 2017), have mainly been reported via immunochemistry, separating 5mC and 5hmC at limited resolution. Although active demethylation is not observed in the maternal pronucleus, it has been reported in the paternal pronucleus of pig (Cao et al., 2014; Lee K. et al., 2014). Both paternal and maternal pronuclei in bovine show 5hmC signal, despite a much less pronounced 5hmC level detected in the maternal pronucleus (Wossidlo et al., 2011; Bakhtari and Ross, 2014). Controversial data have been reported for ovine embryos (Jafarpour et al., 2017; Masala et al., 2017). Jafarpour et al. reported a similar active-demethylation pattern between male and female pronucleus, i.e. strong 5hmC signal in both male and female pronucleus, whereas Masala et al. found 5hmC signal restricted to the paternal pronucleus (Jafarpour et al., 2017; Masala et al., 2017). In horse embryos produced by intracytoplasmic sperm injection (ICSI), both paternal and maternal pronuclei display strong 5hmC signals from PN1 to PN4, yet 5mC levels in both pronuclei are largely unchanged throughout pronuclear development (Heras et al., 2017). Furthermore, these studies reported species-specific *TET* expression in *in vitro* fertilized (IVF) and *in vivo* embryos, as well as aberrant demethylation in somatic cell nuclear transfer (SCNT) embryos (Cao et al., 2014; Zhang S. et al., 2016). The significant differences in staining between species suggest that additional methods that reduce the potential artifacts of immunostaining are essential to bring clarity on the conserved features of DNA demethylation in domestic animal embryos. A recent study used whole-genome, single-base DNA methylation profiling of individual porcine blastocysts, generated *in vivo* or by different IVF processes, and showed abnormally higher methylation levels and lower expression of *Dnmt1, Uhrf1*, and *Dnmt3b* in conventional IVF blastocysts compared to *in vivo* produced embryos (Canovas et al., 2017). These findings warrant further investigation into global demethylation of early embryos of different species and embryos generated by different assisted reproductive techniques.

### Erasure-resistant loci and epigenetic inheritance

During global DNA demethylation in preimplantation embryos, methylation at loci with parental-specific imprints, retrotransposons as well as a portion of differentially methylated CGIs in oocyte-specific and sperm-specific methylated regions are at least partially maintained (Kobayashi et al., 2012; Smith et al., 2012, 2014; Monk, 2015). These methylated regions may be affected by parental life experience as well as *in utero* environment, which could be potential carriers of parental and early life epigenetic information and contribute to epigenetic inheritance. Recent studies show that the sperm methylome can be affected in one or multiple generations by diet (Holland et al., 2016), obesity (Donkin et al., 2016), drug addiction (Feng et al., 2015), stress/traumatic exposure (Franklin et al., 2010; Dias and Ressler, 2014; Gapp et al., 2014b), and toxins in the environment (Skinner et al., 2013; Nilsson and Skinner, 2015). Few studies report altered epigenetic signatures through the female germline, as it is difficult to separate them from the effects of *in utero* variations, including maternal hyperglycemia and gestational diabetes (Vrachnis et al., 2012).

Several factors have been shown to contribute to methylation maintenance at specific loci. Dnmt1 and Dnmt1o, the oocyte-specific form of Dnmt1, contribute to DNA methylation maintenance at imprinted regions (Howell et al., 2001; Ratnam et al., 2002; Hirasawa et al., 2008). Interestingly, similar methylation-protective function of Dnmt1 has been observed in mouse PGCs in the absence of Uhrf1, which is suggested to regulate meiosis-related genes and ensure germline differentiation at the appropriate time (Hargan-Calvopina et al., 2016). Stella/PGC7 protects the maternal genome and ICRs (Imprinting Control Regions) from active demethylation via interaction with histone H3K9me2. In the absence of Stella, loss of 5mC is observed in both pronuclei, accompanied by 5hmC accumulation in the maternal pronucleus (Wossidlo et al., 2011). Trim28/Zfp57 repressive complex which recruits Dnmt1/Uhrf1 as well as Dnmt3a/b is viewed as a non-canonical mechanism of maintaining DNA methylation at ICRs and retrotransposons in preimplantation embryos (Ideraabdullah et al., 2011; Strogantsev et al., 2015). Still, as most of these factors are found at ICRs and retrotransposons, it remains unclear whether similar mechanisms contribute to the maintenance of other methylated regions, especially methylation at genic regions. The Trim28/Zfp57 complex, as well as other Krüppel associated box (KRAB) family members with similar properties as Zfp57, could be potential mediators (Quenneville et al., 2011; Strogantsev et al., 2015). A recent study indicates that loss of maternal Trim28 leads to male-predominant embryo lethality before implantation due to demethylation and activation of *Rbmy1a1* gene, a testis-specific RNA-binding protein involved in alternative mRNA splicing (Sampath Kumar et al., 2017), which emphasizes the significance of methylation-protection mechanisms during early development. Interestingly, higher methylation level of the maternal genome compared to paternal genome is detected at a wide variety of genomic loci in both human embryonic and extra-embryonic lineages from 2-cell to early postimplantation stage, suggesting a potential role of preferential hypermethylation at maternal genome (Zhu et al., 2018). Overall, maintenance of DNA methylation at imprinted genes, retrotransposons and other unidentified genomic regions during epigenetic reprogramming appears to be required for normal development and viability. The identity and function, as well as the maintenance mechanism(s) of these methylated regions, are worth further exploration.

### Epigenetic reprogramming in PGCs—difference between rodent and non-rodent mammals

The maintenance of methylation at imprinted regions is one of the key differences between global demethylation in embryos and in PGCs. Based on DNA methylation and expression profiles obtained from mouse PGCs (mPGCs), global demethylation in PGCs is mediated by two known mechanisms. The first mechanism is replication-dependent demethylation caused by repression of *Dnmt3a/b* and *Uhrf1* expression, which leads to gradual loss of 5mC globally in pre-gonadal PGCs. The second mechanism, which operates mainly in mouse gonadal PGCs, is mediated via Tet1/2 which erase methylation at specific regions such as imprinted regions and germline-specific genes (Seisenberger et al., 2012; Wu and Zhang, 2014). This temporal and mechanistic separation of DNA demethylation in mPGC is suggested to safeguard the timing of germline differentiation (Hargan-Calvopina et al., 2016). The function of Tet1/2 on imprint erasure has been shown in several studies (Dawlaty et al., 2013; Hackett et al., 2013; Yamaguchi et al., 2013a,b). Tet1 may also be involved in regulation of meiosis (Yamaguchi et al., 2012), yet fertility of *Tet1/2* single KO and double male KO mice is overtly normal (Table 1), which questions the functional redundancy and specificity of Tet proteins as well as the necessity of Tet1/2 during PGC demethylation and development. Alternatively, Tet1/2 and 5mC oxidation may be required to “kick-start” the demethylation process (Hahn et al., 2014).

**Table 1:** Key regulators for DNA modifications and their knockout effects.

**Key DNA modification writer and editors**	**Effects of mutants**
*Dnmt1* KO	Embryonic lethality (Liao et al., 2015) Conditional KO in PGCs lead to precocious meiosis in female PGCs and prospermatogonia differentiation in male PGCs (Hargan-Calvopina et al., 2016) Upregulation of post-migratory germ cell-specific genes in the somatic cells of the postimplantation embryo (Maatouk et al., 2006)
*Dnmt3a* KO	Postnatal lethality (Liao et al., 2015)
*Dnmt3b* KO	Embryonic lethality (Liao et al., 2015)
*Dnmt3l* KO	Defects in meiosis (Vlachogiannis et al., 2015)
*Tet1* KO	Viable and fertile (Dawlaty et al., 2011) Defects in imprinting erasure, smaller body size and subfertility (Yamaguchi et al., 2013b) Defects in meiosis (Yamaguchi et al., 2012)
*Tet2* KO	Defects in hematopoietic cell homeostasis(Li et al., 2011) Viable and fertile (Wang et al., 2013)
Maternal *Tet3* KO	Neonatal sublethality (Inoue et al., 2015; Tsukada et al., 2015)
*Tet3* KO	Neonatal lethality (Gu et al., 2011; Wang et al., 2013)
*Tet1/2* DKO	Viable (Wang et al., 2013) Reduction in fertility (Dawlaty et al., 2013)
*Tet* TKO	Impaired morphogenesis and patterning. No headfolds, heart, somites and gut tube formed by E8.5 (Dai et al., 2016)
*Alkbh1* KO	Spermatogenic defects which lead to skewed sex ratio, unilateral defects in eye development and embryonic or postnatal lethality, ranging from E9.5 to P28 (Nordstrand et al., 2010) Defect in placental trophoblast lineage development (Pan et al., 2008) Increased 6mA level in Alkbh1-KO mESCs (Wu et al., 2016)
Fat mass and obesity-associated protein (Fto)	Adipocyte size is smaller in *Fto*-KO mice when compared to WT mice after fed HFD (Church et al., 2009; Fischer et al., 2009; Ronkainen et al., 2015) KD and overexpression of *FTO* in two human cell lines (Jurkat-T, 293T) lead to increased and decreased 6mA contents respectively (Huang et al., 2015)
Thymine DNA glycosylase (TDG) KO	Embryonic lethality (Cortázar et al., 2011; Cortellino et al., 2011)

The overall DNA demethylation dynamics are similar between mouse and human foetal germline. The methylome of gonadal PGCs reaches its lowest level at E13.5 in mouse and week 10 (Wk10) in human, with most promoters and retrotransposons demethylated, yet global gene upregulation does not occur and genome integrity is protected, indicating the global uncoupling of DNA methylation and transcription during epigenetic reprogramming in PGCs. Still, in human PGCs (hPGCs) demethylated promoters of some meiosis-specific genes, such as KRAB-zinc finger proteins (ZFP) and genes of the P-element Induced Wimpy testis (PIWI) pathway, show correlation with increased expression, and are therefore named ‘methylation-sensitive genes’. It is likely that in mammalian PGCs active demethylation at certain regions is required for regulation of germline-specific genes and germline differentiation (Tang et al., 2015; Hargan-Calvopina et al., 2016).

There are key features, at both transcriptional and epigenetic level, that differ between mouse and human PGC development. The temporal and mechanistic separation of DNA demethylation in mPGC may not be conserved in hPGCs. For example, the timing of imprinting erasure is earlier in hPGCs, possibly starting during the migratory phase (before Wk4). This observation is further supported by the upregulation of TET1 and detection of 5hmC in Wk4 hPGCs (Guo et al., 2015; Tang et al., 2015). In contrast, these changes are only observed in early gonadal mPGCs (after E9.5) (Chuva de Sousa Lopes et al., 2008; Guo et al., 2015; Tang et al., 2015; von Meyenn and Reik, 2015). Partial erasure of 5mC coupled with increased level of 5hmC at H19 and GNAS ICRs are also reported in day 4 (d4) and d5 human PGC-like cells (hPGCLCs) produced *in vitro*, which represent pre migratory PGCs (Tang et al., 2015). In contrast, DNA demethylation at *H19* promoter region is only detected after Wk9 in hPGCs (Eguizabal et al., 2016), suggesting that there is significant asynchrony in imprinted gene demethylation in human. Inaccessibility of human embryos at earlier stages prohibits investigations of the *in vivo* demethylation dynamics before week 4, i.e., during hPGC specification and early migration. Thus, developing a model that shares developmental mechanisms with human can be very informative for improving human PGC *in vitro* differentiation systems and for better understanding human PGC development. Our laboratory has developed the pig model, which closely resembles human development during the first three weeks of life. We showed that PGC develop in a similar fashion to what is known in human, and contrasts the process described in mice (Kobayashi et al., 2017).

We showed that global reduction of 5mC and H3K9me2, as well as repression of the epigenetic modifiers UHRF1 and EHMT2/G9a, in newly specified (E14) pre- and early migratory (E16) pig PGCs (Kobayashi et al., 2017). This finding indicates an earlier initiation of epigenetic reprogramming compared to mouse PGCs, but consistent with the timing in hPGCLCs (Kobayashi et al., 2017). Interestingly, along with the reduction of 5mC, TET1 and 5hmC levels are significantly enriched in pre- and early migratory pig PGCs, consistent with largely erased imprinting in Wk4 hPGCs and expression changes of *DNMT3a/b, UHRF1*, and *TET2* in early hPGCLCs (Kobayashi et al., 2017). In agreement with these findings, we showed that demethylation of the imprinted *IGF2R* in male pig PGCs initiates before pig PGCs reach the gonads (Hyldig et al., 2011).

Furthermore, nascent PGC cease to proliferate, suggesting that DNA demethylation is an active process dependent on recruitment of specific factors, such as Tet enzymes in pig PGCs (Kobayashi et al., 2017). These findings show a temporal and mechanistic difference of epigenetic reprogramming between rodents and non-rodent PGCs (Kobayashi et al., 2017). Consistent with these observations in the pig, 5mC and H3K9me2 is also significantly lower in *Cynomolgus* monkey PGCs from as early as ~E17 and E20, respectively (Sasaki et al., 2016), further indicating a difference between PGC development in rodents and non-rodent mammals. Given the similarities between human and porcine embryos at a morphological, transcriptional and epigenetic level, as well as the accessibility of porcine embryos, the pig is a suitable surrogate model for investigating epigenetic reprogramming in human early PGCs and early embryos when combined with *in vitro* models for human embryonic development.

Although the methylated regions maintained during the reprogramming wave in preimplantation embryos are potential carriers of parental ‘epimutations’, they also need to ‘escape’ the global demethylation in PGCs to be able to contribute to transgenerational epigenetic inheritance. The idea of transgenerational inheritance of epigenetic states in mammal was first proposed about 30 years ago (Holliday, 1987; Laird, 1987). Although there is no definitive proof to show that DNA methylation marks can be maintained in germline and inherited across multiple generations in mammals, recent evidence suggests that DNA methylation at some genomic loci can escape both waves of epigenetic reprogramming (Seisenberger et al., 2012; Hackett et al., 2013; Guo et al., 2015; Tang et al., 2015). These regions are termed ‘escapees’, and a group of these escapees are repeat-poor, single copy genes which are associated with metabolic and neurological disorders (Tang et al., 2015).

Overall, redundant mechanisms have evolved to act in parallel for the efficient erasure of epigenetic marks in early embryos and PGCs, yet several mechanisms also exist to protect methylation at specific genomic regions, which could contribute to epigenetic inheritance. These demethylation and methylation-protection mechanisms may differ between rodent and non-rodent mammals given the different demethylation dynamics reported, suggesting recent and presumably ongoing evolutionary selection processes.

## Specific features of 5hmC

### 5hmC as a regulatory marker

5hmC is mainly viewed as an intermediate product of active demethylation and is rapidly removed during replication (Inoue and Zhang, 2011) or further oxidized to 5fC and 5caC. However, the non-reciprocal pattern between 5mC and 5hmC and the dynamics of hydroxymethylation observed during late oogenesis and early embryo development in multiple mammalian species suggest an additional biological role for 5hmC in early development (Masala et al., 2017). The enrichment of 5hmC and TET1/2 before and during pig PGC specification and early hPGCLC development also indicates the necessity of hydroxymethylation in non-rodent PGC development. The stability and abundance of 5hmC observed in mESCs further supports its biological significance (Bachman et al., 2014; Zhao et al., 2014). Still, the contribution of Tet proteins and 5hmC to pluripotency and differentiation is controversial. In mouse preimplantation embryos and mESC Tet functions are linked with pluripotency genes. For example, Tet3 deficiency in oocytes hinders DNA demethylation of the paternal *Oct4* and *Nanog* genes in early embryos, and leads to delayed activation of a paternally derived *Oct4* transgene (Gu et al., 2011; Guo F. et al., 2014). During somatic cell reprogramming of mouse induced-pluripotent cells (iPSCs), Tet1 and Tet2 interact with Nanog and redundantly enhance Nanog-mediated reprogramming to pluripotency. However, recent data indicate that Tet1 and Tet2 have opposing effects during the establishment of naïve pluripotency, which is the ‘ground’ state of pluripotency in naïve epiblast in mice (Fidalgo et al., 2016). Tet1 partners with Zfp281 for establishing and maintaining primed pluripotency, and thereby also negatively regulates *Tet2* expression. Ectopic expression of *Tet2* alone in mESCs, but not *Tet1* or *Tet3*, promotes reprogramming to naïve pluripotency, whereas knockdown (KD) of *Tet2* leads to significantly reduced 5hmC at naïve pluripotency genes and compromised reprogramming to naïve state (Fidalgo et al., 2016). Other pluripotency genes, such as *Oct4* (Piccolo et al., 2013) and *Lin28* (Jiang and Baltimore, 2016; Tan and Yeo, 2016; Zeng et al., 2016), have also been related with Tet recruitment and its enzymatic activity.

It is largely unclear in which regions Tet-mediated active demethylation and 5hmC have transcriptional-regulating roles during pre-implantation embryo and PGC development. A recent study suggests that the enzymatic activity of Tet proteins, specifically of Tet3, is required for antagonizing Dnmt3a/b functions and regulating Lefty–Nodal signaling during gastrulation (Dai et al., 2016). Still, it remains unclear whether the requirement of Tet-mediated active demethylation is due to (1) protection of *Lefty* regions from passive demethylation and/or (2) a specific role of 5hmC at these loci. The transcriptional regulatory function of 5hmC may depend on different binding properties compared to 5mC or unmethylated C, leading to a specific protein-binding profile at 5hmC-enriched regions. For example, binding of MeCP2, Mbd3, and Uhrf1 to 5hmC was detected *in vitro* and in genomic DNA of mESCs and in the nervous system (Frauer et al., 2011; Yildirim et al., 2011; Mellén et al., 2012; Iurlaro et al., 2013; Chen et al., 2014). However, further studies suggest no binding preference for 5hmC and therefore these three proteins are unlikely to be 5hmC-specific readers (Hashimoto et al., 2012; Mellén et al., 2012; Baubec et al., 2013; Spruijt et al., 2013; Cui and Irudayaraj, 2015; Gabel et al., 2015; Hainer et al., 2016). Additional uncharacterized proteins have been identified as 5hmC readers by Spruijt et al. (2013). For example, Uhrf2, a close relative of Uhrf1, shows strong preference for 5hmC and is considered a bona fide 5hmC reader (Spruijt et al., 2013). A further study reveals the structural basis of Uhrf2's preference for 5hmC (Zhou et al., 2014). KO of *Uhrf2* in mice leads to reduction of 5hmC in the cortex and the hippocampus, along with abnormal expression of neuronal-related genes and defects in spatial learning and memory (Chen R. et al., 2017). Another 5hmC-interacting protein identified is C3ORF37/Hmces/Srap1, a eukaryotic member of the SRAP superfamily (Aravind et al., 2013) that functions as an eraser of 5hmC, *in vitro* and in mESCs. *Srap1* KO causes sub-lethality in pre-implantation blastocysts, but the mutant mice are fertile, suggesting an unaffected PGC development (Kweon et al., 2017) and different reprogramming mechanisms between preimplantation embryo and PGCs.

Current genome-wide 5hmC detection techniques are not ideal for studies in early embryos or PGCs due to the amount of starting material required (Yu et al., 2012; Petterson et al., 2014; Wu et al., 2014), as well as other technical limitations such as low genomic coverage (Sun et al., 2013; Han et al., 2016). Still, extensive research in mESCs and several other cell lines has shed some light on potential roles of 5hmC (Wu and Zhang, 2011; Xu et al., 2011; Cimmino and Aifantis, 2017). The correlation between 5hmC and gene expression appears to be quite diverse and spatiotemporal-dependent. The genomic 5hmC pattern itself is also highly dynamic, which has been correlated with lineage-commitment gene expression and tissue-specific biological processes (Nestor et al., 2012; Lin et al., 2017; Ponnaluri et al., 2017). In general, 5hmC enrichment on gene bodies is linked with gene activation (Wu and Zhang, 2011; Guo et al., 2016; Lin et al., 2017; Ponnaluri et al., 2017). However, the effect of 5hmC enrichment on gene bodies has also been described as ‘mildly inhibitory’, as it correlates with modest gene up-regulation (Neri et al., 2013). 5hmC on promoters and enhancers correlates with both active (Ficz et al., 2011; Stroud et al., 2011; Deplus et al., 2013) and repressed genes (Williams et al., 2011; Wu and Zhang, 2011; Choi et al., 2014; Kim et al., 2014; Zhang X. et al., 2016). Genomic 5hmC distribution is largely dependent on the activities of Tet proteins, which preferentially bind to promoters and gene bodies, but also enhancers with affinity that correlates with CpG density (Stroud et al., 2011; Williams et al., 2011; Branco et al., 2012). Tet1 and Tet3 contain a DNA binding CxxC domain which Tet2 lacks, yet the functional redundancy and specificity of Tets are still unresolved. Apart from potentially different contributions to pluripotency, in mouse embryo, Tet1, 2, and 3 also show distinct expression profiles, suggesting that the requirement of Tet activities are highly context-dependent (Khoueiry et al., 2017). Also, Tet1, and Tet2 show different localization in the genome, albeit with some overlap. Tet1 is suggested to be mainly responsible for 5hmC generation at promoters and some enhancers, while Tet2 with 5hmC is present along gene bodies (Huang et al., 2014). Reduced 5hmC and increased 5mC are observed at enhancers in Tet2^−/−^ mESCs, which leads to reduced activities in a subset of enhancers and gene activation during mESC differentiation to neuronal fate, suggesting that Tet1 plays a role in the oxidation of some enhancers (Hon et al., 2014). The role of Tet proteins, especially around transcription start sites (TSSs), could be independent of their catalytic function (Williams et al., 2011), and more related to their interaction/recruitment with other proteins such as O-Linked N-Acetylglucosamine (GlcNAc) Transferase (OGT) and Polycomb repressive complex 2 (PRC2) at CpG-rich regions (Wu et al., 2011; Xu et al., 2011; Chen et al., 2013; Deplus et al., 2013; Neri et al., 2013; Vella et al., 2013). This may in part explain the active and repressive roles of 5hmC. It is also possible that the genomic context of 5hmC contribute to the regulation of specific genes during development and differentiation by modulating chromatin accessibility (Ruzov et al., 2011). In foetal germ cells, genes with intermediate 5hmC levels of promoter regions are more likely to display higher chromatin accessibility and also have higher gene expression (Guo et al., 2016). Tet activity on insulators may also regulate gene expression by altering chromosomal architecture (Marina et al., 2016). 5hmC and Tet activities may affect other epigenetic markers or modifiers. For example, the correlation between 5hmC and bivalency, a poised epigenetic state defined by co-existence of active marker H3Lys4-trimethylation (H3K4me3) and repressive H3Lys27-trimethylation (H3K27me3), characteristic of pluripotent cells, has been reported in multiple studies (Pastor et al., 2011; Gao et al., 2013; Neri et al., 2013; Kong et al., 2016).

### The 5mC/5hmC asymmetry

5mC on DNA strands is considered symmetrical and is maintained by Dnmt1/Uhrf1 through replication, whereas Tet-mediated active demethylation from 5mC is the only known origin of 5hmC, with no known mechanism for maintenance through cell proliferation. Interestingly, asymmetric distribution and inheritance of 5hmC on complementary CpG sites between Watson and Crick strands (CpG dyads) is observed during mouse early embryogenesis (Guo H. et al., 2014; Mooijman et al., 2016) and in adult stem cells (Huh et al., 2013). This asymmetry is suggested to be mostly 5mC/5hmC, but unlikely to be C/5hmC or C/5mC at single CpG sites (Zhao et al., 2014). A high 5mC/5hmC abundance at CpG dyads was also found in mESCs using a novel detection technique, suggesting potential biological functions for the 5mC/5hmC modification status (Song et al., 2016). *In vitro* assays indicate that Uhrf1 and Dnmt3A can recognize hemi-hydroxymethylated substrates, whereas Dnmt1 cannot (Frauer et al., 2011). Further investigation of the activities of Tet proteins and Dnmt proteins *in vivo* may reveal if the 5mC/5hmC asymmetry could be established by preferential oxidization, maintenance or *de novo* establishment of methylation on only one strand of 5mC/5mCpGs.

## 5fC and 5caC: intermediates of demethylation or regulatory markers?

5fC and 5caC were considered as transient intermediate products of Tet-mediated active demethylation. During this process these modifications are removed by either passive dilution during replication, or are excised by thymine DNA glycosylase (Tdg) followed with base excision repair (BER) for replacement of an unmodified cytosine (He X.-J. et al., 2011; Maiti and Drohat, 2011). A recent study suggests that C3ORF37/Hmces/Srap1 functions as an eraser for 5fC and 5caC in mESCs (Kweon et al., 2017). The presence of 5fC (0.06–0.6% of 5mC) and 5caC (0.01% of 5mC) in the mammalian genome is extremely low compared to 5hmC and 5mC (Ito et al., 2011; Pfaffeneder et al., 2011; Booth et al., 2014; Bachman et al., 2015). One potential explanation for this low occurrence is the preference of Tet for 5mC over 5hmC/5fC (Hu et al., 2015). Administration of stable isotope [*methyl*-^13^CD_3_]L-methionine tracked with liquid chromatography coupled to mass spectrometry/high resolution mass spectrometry (LC-MS/HRMS) further indicates that 5fC is a stable DNA modification in mESCs (Bachman et al., 2015). The stability of 5fC is also supported by another study indicating that the majority of 5fC at CpG sites is not removed via passive dilution during the gamete to 4-cell stage progression of the mouse embryo (Guo H. et al., 2014). However, neither 5fC nor 5caC were detected by mass spectrometry 8 h post-fertilization (Okamoto et al., 2016). Although 5fC and 5caC are considered relatively stable during mouse PGC development (E9.5 to E12.5), both epigenetic marks are not detected in human PGCs at comparable stages (Wk4-9) (Yamaguchi et al., 2013a; Tang et al., 2015).

### Distribution of 5fC and 5caC

Reports indicate that 5fC and 5caC may stably exist at specific genomic regions, with distinct effects on gene expression. Several studies reported that 5fC/5caC overlap with H3K4me1 marked regions which are associated with active or poised transcription (Shen et al., 2013; Song et al., 2013; Wu et al., 2014). In *Tdg* KO mESCs, promoters with silent or bivalent histone marks, as well as active enhancers and pluripotency TF-binding sites are enriched for ectopic 5fC/5caC signals, indicating potential roles of 5fC/5caC and targeted demethylation mediated via Tdg in mESCs (Shen et al., 2013). As ectopic 5fC/5caC are also enriched at promoters of genes with low-to-medium expression levels, they are considered as ‘mildly repressive’ (Shen et al., 2013). Poised enhancers show enriched 5fC in mESCs, and p300-based activation of enhancers coincides with an increase of 5fC in *Tdg*-KO mESCs (Song et al., 2013). Single-base mapping of 5fC and 5caC further confirm their enrichment at active enhancers and reveal differential genomic profiles for 5fC and 5caC, with 5fC more frequently present in exons and promoter regions, and 5caC more frequently present in intronic regions. 5fC and 5caC are also detected with different sequence preference and binding sites of pluripotent genes. The gradient of 5mC, 5hmC, 5fC, and 5caC further indicates the correlation between Tet-mediate demethylation and the activation of regulatory elements in mESCs (Lu et al., 2015). Apart from the enrichment of 5fC on active enhancer regions, single-based 5fC profiles in multiple tissues also indicates clear tissue specificity of 5fC distribution (Iurlaro et al., 2016). More recently, 5fC was profiled in mouse early embryos in single cells at single base resolution, revealing stage-specific and parental-specific dynamics of 5fC. Interestingly, a critical set of developmental and metabolic related genes shows 5fC enrichment on promoters before gene upregulation (Zhu et al., 2017), which is also supported by the finding of transient 5caC accumulation at promoter regions preceding gene expression during lineage specification and differentiation (Lewis et al., 2017). These results highlight a fine balance between Tet oxidation and Tdg excision, suggesting potential roles for 5fC/5caC and Tet/Tdg-mediated demethylation in embryonic development as well as tissue-specific processes. However, removal of 5fC/5caC is largely independent of Tdg in both pre-implantation embryos (Guo F. et al., 2014; Guo H. et al., 2014; Zhu et al., 2017) and in mammalian PGCs (Yamaguchi et al., 2013a; Tang et al., 2015). This raises questions about the stability of 5fC/5caC over replication-dependent passive dilution and suggests the existence of other removal mechanisms, such as the recently identified Srap nuclease (Kweon et al., 2017).

### Specific functions and protein binding partners for 5fC and 5caC

Several studies have focused on the potential mechanisms underlying the correlations between 5fC/5caC and the biological processes mentioned above (Spruijt et al., 2013; Song and Pfeifer, 2016). *In vitro* assays indicate that 5fC and 5caC lead to altered transcriptional properties, including increased backtracking, increased pausing, and reduced fidelity of mammalian RNA polymerase II (Pol II) in nucleotide incorporation (Kellinger et al., 2012). Structural and biochemical analysis show that specific hydrogen bonds are formed between the 5-carboxyl group of 5caC and the conserved epi-DNA recognition loop in Pol II. Comparison between *Tdg*-KO and wild-type mESCs shows a clear reduction of Pol II elongation in *Tdg*-KO cells, suggesting that 5fC/5caC at gene bodies function as another layer of fine-tuning of Pol II transcription elongation *in vivo* (Wang et al., 2015). 5fC was reported to alter the DNA double helix structure which is likely to lead to helical unwinding *in vitro* (Raiber et al., 2015). However, compared to equivalent unmodified duplexes and sequence variants 5fC does not significantly affect the structure of the DNA double helix in a crystalline state (Hardwick et al., 2017). The alteration described by Raiber et al. (2015) is not unique to 5fC modified sequences, and therefore is unlikely to be the basis for 5fC recognition (Hardwick et al., 2017). Still, 5fC may trigger biological events by affecting the mechanical properties of DNA, for example, by increasing DNA flexibility (Ngo et al., 2016). Ngo et al. also show that 5hmC enhances flexibility, but is less effective compared to 5fC, while 5mC reduces flexibility and 5caC is similar to unmodified cytosine in terms of structural qualities. DNA flexibility positively affects nucleosome stability, which may link 5fC with its regulatory role of gene expression at enhancer regions (Ngo et al., 2015, 2016). Protein binding affinity, which includes binding to histone octamers, is also affected by DNA flexibility (Peters and Maher, 2010). Screens for binding proteins indicates that there are more proteins with a strong preference for 5fC compared to 5hmC, revealing the existence of potential ‘readers’ for 5fC which are involved in several biological processes including transcription regulation, chromatin regulation and DNA repair (Iurlaro et al., 2013).

Overall, although the stability of 5fC and 5caC requires validation, current data suggests that potential functions of 5fC and 5caC as well as their modifiers and readers are worth further investigations, especially in early embryos and PGCs with ongoing global DNA demethylation. Newly developed techniques which are cost-effective (Neri et al., 2016) and require small numbers of cells (Zhu et al., 2017) will be helpful in future studies to resolve these issues. Different biochemical characteristic of 5hmC/5fC/5caC and their biological roles, if validated, represent additional regulatory layers during active removal of 5mC.

### 6mA

6mA, the most prevalent DNA modification in prokaryotes is used by bacteria to regulate the gene expression program related to DNA repair (Messer and Noyer-Weidner, 1988), virulence (Sarnacki et al., 2013) and cellular defense (Zaleski et al., 2005). Interestingly, 6mA is considered a novel epigenetic mark in eukaryotes, as it has been reported only recently in several fungi (Mondo et al., 2017), ciliates (*Tetrahymena thermophila*) (Wang Y. et al., 2017), green algae (*Chlamydomonas Reinhardtii*) (Fu et al., 2015), nematodes (*Caenorhabditis elegans*) (Greer et al., 2015), fruit fly (*Drosophila melanogaster*) (Luo et al., 2015; Zhang et al., 2015; O'Brown and Greer, 2016), zebrafish (*Danio rerio*) (Liu J. et al., 2016), african clawed frog (*Xenopus laevis*) (Koziol et al., 2016), and mammals (Koziol et al., 2016; Liu J. et al., 2016). Potential biological functions of 6mA in eukaryotes include regulation of gene transcription (Fu et al., 2015; Wu et al., 2016; Mondo et al., 2017), cell cycle, transposon activities (Zhang et al., 2015; Liu J. et al., 2016), nucleosome positioning (Mondo et al., 2017), as well as ribosomal DNA preservation (Blackburn et al., 1983; Wang Y. et al., 2017) and crosstalk with histone modification (H3K4me2), which contributes to transgenerational epigenetic inheritance (Greer et al., 2015). An *in vitro* study further reported a specific pausing effect of 6mA on yeast RNA polymerase II (pol II) (Wang W. et al., 2017). However, the epigenome of *C. elegans* and *D. melanogaster* could be largely different to that of mammals, as they are nearly devoid of 5mC (Capuano et al., 2014; Greer et al., 2015; Zhang et al., 2015). Therefore, a potentially highly divergent distribution and functions of 6mA occurring in these organisms may not be conserved in vertebrates. Indeed, 6mA level in vertebrates is extremely low in various examined tissues, from 0.00009% 6mA/A detected in *X. laevis* tissues (Koziol et al., 2016) to the highest of ~0.1–0.2% 6mA/A during early embryogenesis of zebrafish and pig (Liu J. et al., 2016). Also, the distribution of 6mA seems highly species-specific. In *X. laevis* tissues and mouse tissues, the peaks of 6mA are mainly found in non-genic regions, while only a small set of genes contains 6mA (Koziol et al., 2016). 6mA at genic regions is more frequent at introns and is nearly depleted from TSSs and exons, which is in contrast to the enrichment of 6mA at or following TSS in *Chlamydomonas* and *Drosophila* genomes, or the even distribution of 6mA in *C. elegans* genome. 6mA is also enriched at intergenic over promoter regions in zebrafish oocytes and early embryos. However, in contrast to the above result in *X. laevis* and mouse tissue, 6mA DNA immunoprecipitation sequencing (DIP-seq) revealed slight 6mA enrichment after TSSs and at exons instead of introns in zebrafish (Liu J. et al., 2016). Most interestingly, in several eukaryotic species, a large portion of 6mA detection peaks are within repetitive elements (REs), especially in simple repeat regions. Based on these findings, significant motif sequences have been identified in simple repeats and other REs (Liu J. et al., 2016; Wu et al., 2016).

Other distinct features of 6mA have also been revealed, indicating that despite the overall low level of 6mA, this particular DNA modification could be very dynamic and functionally important in vertebrates. Half of the 6mA sites in *X. laevis* tissues and mouse kidney seem to be tissue-specific, and GO term analysis indicates that genes modified by 6mA are linked to different pathways, which could indicate either a tissue-specific role, or a species-specific role of 6mA modification (Koziol et al., 2016). Abundance of 6mA in oocytes and early embryo of zebrafish and pig (0.1–0.2% 6mA/A) (Liu J. et al., 2016) suggests a specific role of 6mA in early embryo development of 6mA. Interestingly, cancer cells and tissues display significantly decreased 6mA levels when compared to normal cells and tissues, whereas cultured cells show hundred-fold elevated 6mA levels compared to *in vivo* tissues, in both human and mouse (Liang et al., 2016). These results suggest that 6mA abundance could rapidly alter during changes of cell identity. KO of *Alkbh1* in mESC, a possible 6mA demethylase, revealed 550 downregulated genes which are enriched in development and lineage specification factors. In addition, imbalanced cell fate decisions during differentiation further suggest a critical role of 6mA in early development. In *Alkbh1* KO mESCs, 6mA was found significantly enriched at the retained 5′ UTR and open reading frame 1 (ORF1) regions of full-length, evolutionarily young long interspersed nuclear element 1 (L1/LINE-1), along with elevated 5mC levels compared to wild-type mESCs. Further analysis indicated that active transposable elements–of the LINE-1 class - could be repressed by 6mA, and thereby function as a silencing center for neighboring genes during differentiation (Wu et al., 2016), which contradicts previous suggestions that 6mA may be an active mark for gene expression in lower eukaryotes (Fu et al., 2015; Mondo et al., 2017). Extra deposition of 6mA on the X chromosome in *Alkbh1* KO mESCs is also reported to have a long-lasting, repressive effect, which may partially explain the imbalanced cell fate decision observed during *Alkbh1* KO mESCs differentiation. However, the observation of *Alkbh1* as a 6mA demethylase was not supported by another study using *Alkbh1* knockout mESCs and mouse embryonic fibroblasts (MEFs) as well as *in vitro* biochemical assays (Liu F. et al., 2016). Instead, Liu et al. suggest that *Alkbh1* is a demethylase of m1A (N1-methyladenosine on RNA), which affects the initiator methionyl-transfer RNA (tRNA^iMet^) level and translation initiation in mammalian cell lines. Moreover, the existence of 6mA in mESCs and mouse tissues was recently questioned by a highly sensitive LC-MS method; in parallel, the same approach successfully detected 6mA at a level of 0.7% 6mA/A in *Chlamydomonas Reinhardtii* genome (Schiffers et al., 2017). The authors suggested that the previous sequencing-derived data of 6mA in mESCs might be caused by degraded bacterial DNA which provides the 6mA nucleoside incorporated into mESC DNA (Schiffers et al., 2017). Therefore, further profiling and validation of DNA and RNA modification in wild type and *Alkbh1* KO mammalian cell lines with different detection methods and contamination-eliminating strategies may help solve the controversy. In human cell lines, Fat mass and obesity-associated protein (FTO), which has previously been identified as a demethylase for N6-methadenine on RNAs (m6A) both *in vivo* (Shen et al., 2015) and *in vitro* (Jia et al., 2011), is suggested as another 6mA demethylase (Huang et al., 2015). Interestingly, the level of N6-methadenine on both DNA and RNA (Klungland and Dahl, 2014) seems to correlate with cellular fat mass and may be linked to obesity and type 2 diabetes (Huang et al., 2015).

Overall, the presence of 6mA in eukaryotic genomes, especially lower eukaryotes, is evident, with divergent genomic distribution and functions observed in different eukaryotic species. Thus, it is worth further exploring novel modifications such as 6mA. If 6mA truly has significant biological functions, the specificity of 6mA establishment, removal and readout will be crucial for understanding its roles. Current data indicate a lack, or insufficient, consensus motifs for 6mA establishment in vertebrate genomes based on *in silico* analysis (Koziol et al., 2016). It is possible that the ‘modifiers’ do not recognize specific sequences, but are recruited by different 6mA-binding proteins. Also, interesting features of 6mA observed in lower eukaryotes, such as co-regulation with active histone mark H3K4me2 and involvement in transgenerational inheritance (Greer et al., 2015), have not been investigated in vertebrates.

It has been suggested that, in general, the levels of 6mA and 5mC have an inverse relationship in organisms throughout the tree of life (O'Brown and Greer, 2016; Mondo et al., 2017). There seems to be an evolutionary shift from 6mA to 5mC as the major epigenetic mark. It is worth mentioning that the Tet homolog is identified as 6mA demethylase in *Drosophila* (Zhang et al., 2015), which supports such an evolutionary shift and also suggests the possibility of an inverse correlation between 5mC and 6mA in eukaryotes (Breiling and Lyko, 2015). 6mA, unlike 5mC, does not tend towards spontaneous mutations and therefore could be advantageous for genomic stability compared to 5mC. Interestingly, the timing of 6mA enrichment after fertilization (Liu J. et al., 2016) overlaps with the timing of global demethylation in zebrafish (Potok et al., 2013) and pig (Cao et al., 2014), in spite of the overall different dynamics between zebrafish and mammalian epigenetic reprogramming (O'Neill, 2013; Luo and He, 2017). It would be interesting to investigate if the enrichment of 6mA is truly correlated with genome-wide demethylation and plays repressive roles complementary to 5mC on regions such as REs in preimplantation embryos and PGCs (Liu F. et al., 2016; Luo and He, 2017). Whether spatiotemporal changes of 6mA levels occur under abnormal circumstances, such as the state of obesity and type 2 diabetes, is also worth exploring. However, the existence of 6mA in animals and whether 6mA is abundant or stable enough to elicit biological changes in vertebrate genomes still remains questionable.

## 5mC at non-CpG sites

Although DNA methylation (5mC) is predominantly found on CpG sites in mammalian genomes, non-random distribution of non-CpG methylation is present in the mouse and human genome and has been studied for their biological relevance for over a decade (Pinney, 2014; He and Ecker, 2015). Non-CpG methylation is present in most tissues (Schultz et al., 2015), but only prevalent in a few cell types and tissues such as ESCs (Ramsahoye et al., 2000; Lister et al., 2009; Ziller et al., 2011), brain (Guo J. U. et al., 2014; Kinde et al., 2015), and oocytes (Tomizawa et al., 2011; Shirane et al., 2013). Non-CpG methylation is also accumulated in mitotically arrested prospermatogonia (Kobayashi et al., 2013), but is largely lost after the resumption of mitosis (Ichiyanagi et al., 2013), which is consistent with the lack of maintenance mechanism for non-CpG methylation. Non-CpG methylation is almost exclusively established by Dnmt3a/b and requires the presence of Dnmt3L (Arand et al., 2012; Vlachogiannis et al., 2015). As Dnmt1 generally does not contribute to non-CpG methylation, the distribution is asymmetrical between the two strands of individual DNA molecules. 5hmC on non-CpG sites is very rare, even in brain (Lister et al., 2013) and ESCs (Yu et al., 2012), suggesting that Tet-mediated demethylation is not required for removal of non-CpG methylation.

Non-CpG methylation has been suggested to be a by-product of Dnmt activity due to its strong link to neighboring CpG methylation (Ziller et al., 2011; Arand et al., 2012). However, motif analysis suggests that non-CpG methylation does have tissue-specific site preferences in the genome (Lister et al., 2009, 2013; Vlachogiannis et al., 2015). Apart from tissue-specific distribution, non-CpG methylation on promoters, enhancers and gene bodies also have been associated with transcriptional repression (Guo J. U. et al., 2014; Zhang et al., 2017) and protein binding activities (Barrès et al., 2009; Guo J. U. et al., 2014; Sperlazza et al., 2017). Aberrant non-CpG methylation patterns were shown in pluripotent cells (Lister et al., 2011; Ma et al., 2014), yet the correlation between non-CpG methylation and pluripotent states is largely unknown (Patil et al., 2014). Also, the prevalence of non-CpG methylation in oocytes is worth exploring, as maternally-derived methylation could have functions during early embryo development and therefore have the potential as carrier of epigenetic information (Branco et al., 2016).

## 5hmU

Apart from modifications on cytosine and adenine, oxidized thymine (T) nucleobases such as 5-hydroxymethyluracil (5hmU) are also found in mammalian cells. 5hmU was considered as a product of oxidative lesions (Bjelland et al., 1995) and mainly precursors of Base J (β-D-Glucopyranosyloxymethyluracil) in eukaryotic kinetoplastid flagellates (van Luenen et al., 2012; Reynolds et al., 2014; Kawasaki et al., 2017). A recent study reports the presence of 5hmU in human, rat and porcine tissues at relatively stable levels (Gackowski et al., 2015). In mESCs, the majority of 5hmU is generated by Tet-catalyzed oxidation of thymine instead of reactive oxygen species (ROS)-induced oxidation. Compared to adult somatic tissue, 5hmU levels are elevated in mESCs and are found in amounts that are comparable to 5caC. 5hmU levels peak between 8 and 16 hours after naïve mESCs begin differentiation towards post-implantation epiblast, suggesting 5hmU may be largely generated during epigenetic reprogramming (Pfaffeneder et al., 2014; Robertson et al., 2014). Although previous studies suggest that 5hmU can be produced via deamination of 5hmC by the Aid (activation-induced cytidine deaminase) / Apobec (apolipoprotein B mRNA-editing catalytic polypeptides) family of enzymes (Cortellino et al., 2011; Guo et al., 2011), isotope tracing experiments in mESCs suggests that 5hmC is not a 5hmU precursor (Pfaffeneder et al., 2014). *In vitro* studies also suggest that 5hmC is an unlikely substrate for enzymes of the Aid/Apobec family (Nabel et al., 2012; Rangam et al., 2012; Budzko et al., 2017). A set of transcription factors and chromatin remodeling factors in nuclear extracts from mESCs have been identified as specific readers of 5hmU sites (Pfaffeneder et al., 2014). The Tet-mediated elevated levels of 5hmU during mESC differentiation and the specific readers identified suggests that 5hmU may not simply be an oxidative lesion but have regulatory roles during early embryonic development. Further investigations of the 5hmU dynamics in mammalian model organisms are warranted (Olinski et al., 2016).

## Novel modification and disease

As DNA methylation (5mC) is a well-recognized epigenetic regulator for gene expression, abnormality of 5mC profiles in various diseases (Robertson, 2005), including cancer (Esteller, 2008), aging-related disease (Gassen et al., 2017), cardiovascular diseases (Chistiakov et al., 2017), metabolic disorders (Wahl et al., 2017), reproductive disorders (Ho et al., 2017), and mental/neurological disorders (Nestler et al., 2016), have also been extensively studied and documented. Many of those diseases and their abnormal 5mC profiles may be attributable to early life experiences, suggesting the involvement of DNA methylation in the Developmental Origins of Health and Disease (DOHaD) paradigm (Leenen et al., 2016). Recent studies have also revealed potential roles of 5hmC in disease. Reduced 5hmC levels have been reported in a variety of cancers (Yang et al., 2013; Ficz and Gribben, 2014; Pfeifer et al., 2014; Rasmussen and Helin, 2016), which makes loss of 5hmC a potential hallmark of cancer (Chen Z. et al., 2017). Still, the biological consequence of the universal loss of 5hmC in tumorigenesis is not clear. Aberrant 5hmC pattern has also be observed in aging-related diseases (López et al., 2017), and age-related enrichment of 5hmC at specific loci has been observed in neural system (Szulwach et al., 2011; Chen et al., 2012). 5hmC has also been linked with neurological/mental disorders such as depression (Tseng et al., 2014; Gross et al., 2017), autism (Papale et al., 2015; Madrid et al., 2016), and Parkinson's disease (Stöger et al., 2017), as well as developmental disorders such as fragile X syndrome (Brasa et al., 2016; Esanov et al., 2016). On the other hand, disease-related profiles of more recently discovered DNA modifications remain largely unknown. For example, decreased 6mA level has been observed in cancers (Liang et al., 2016) and type 2 diabetes (Huang et al., 2015), suggesting that investigation of the role of 6mA in those diseases is needed.

## Future investigation and challenges

All DNA modifications currently studied in mammalian genomes display their most dynamic profiles during epigenetic reprogramming in early development. Therefore, preimplantation embryos and PGCs, as well as their corresponding *in vitro* cell lines where epigenetic reprogramming also occurs, provide tractable systems for studying novel DNA modifications. Here we list a few aspects of novel DNA modifications that are worth exploring further:

### Genome-wide distribution of novel modification during early development and its potential of epigenetic inheritance

Genomic distribution of novel modifications may show specific spatiotemporal patterns during early development. Combined with other omics data, having a clear understanding of how these patterns are regulated may further our knowledge of the potential roles of these marks in development and inheritance.

**Figure 1:**
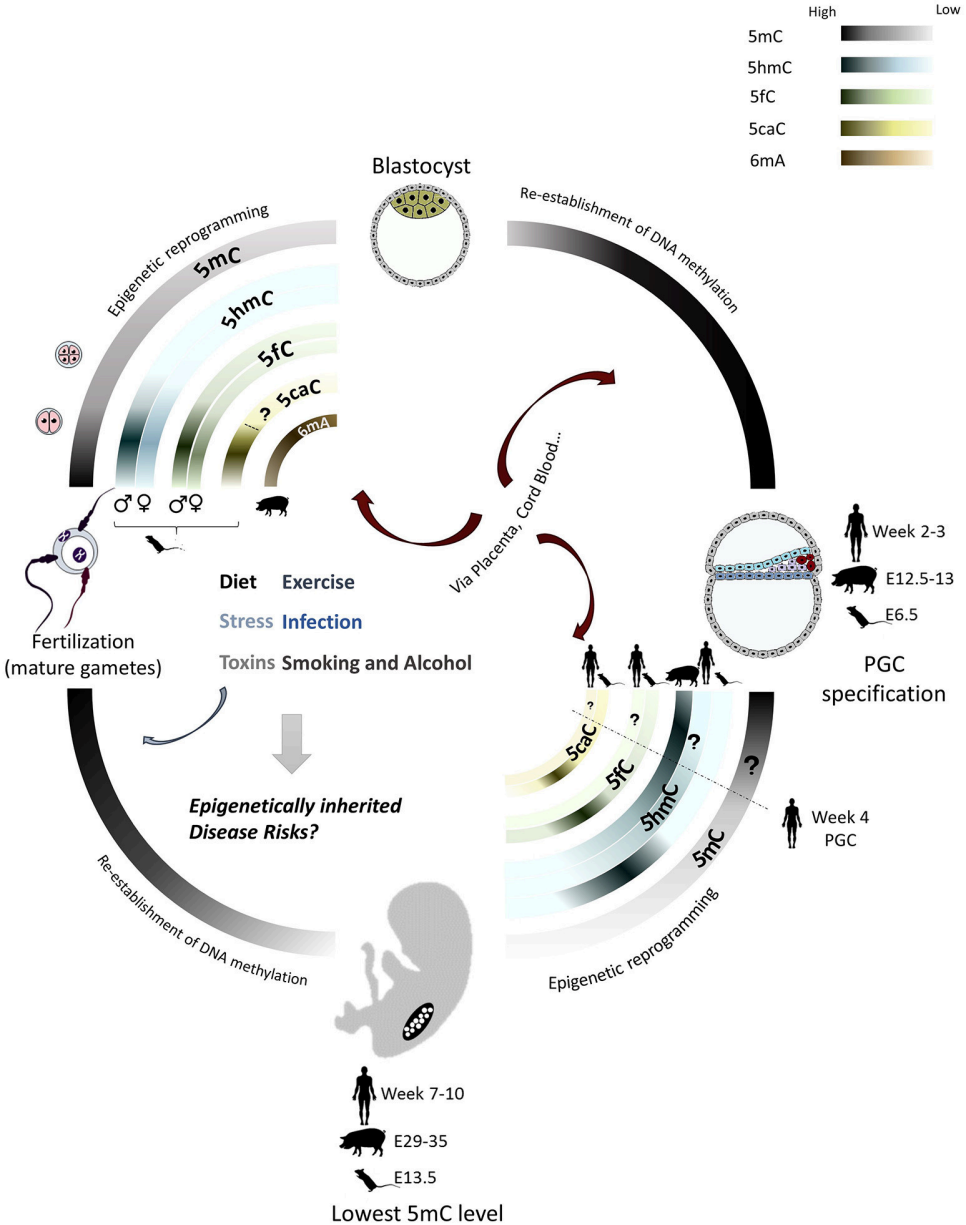
DNA methylation in the mammalian lifecycle DNA methylation profiles show differences in the lifecycle of different mammals. They are most dynamic during the epigenetic reprogramming in preimplantation embryo and primordial germ cells (PGCs). 5mC is largely erased during both epigenetic reprogramming events in the pre-implantation embryo and germ cells and subsequently re-established. Methylation reprogramming in PGCs is more extensive than in early embryo, including demethylation of imprinted genes and repetitive elements. 5hmC, 5fC and 5caC are the oxidized products of TET-mediated active demethylation. Timing and extent of TET-mediated active demethylation differ between genders in pre-implantation embryos and in PGCs of different species. 6mA is found enriched in pig oocytes and early embryos. The epigenome is much more vulnerable to environmental influences, especially during the extensive remodeling phases, which may lead to abnormal epigenetic states in the embryo and germline that contribute to future development and inheritance of disease.

Considering the low abundance of some of the DNA modifications, cross-validation by multiple strategies is required; this currently limits studies in rare cell types and tissues. For example, bisulfite sequencing is unable to distinguish between 5mC and 5hmC, or unmethylated cytosines from 5fC and 5caC, which may lead to misinterpretation of 5mC profiles and dynamics of active demethylation. Advanced techniques with improved sensitivity and specificity would allow genome-wide profiling of rare epigenetic markers in small numbers of specific cells, including PGCs, preimplantation embryos and sub-populations of cancer cells *in vivo*. This will bring new understanding about the biological role of those novel modifications. Also, comparing genome-wide distribution in different stages of the lifecycle will enable the distinction between genomic regions that maintain stable marks from those that are susceptible to environmental influences. Such high-resolution profiling of modifications will enhance our understanding of the potential role of different DNA modifications in epigenetic inheritance (Figure 1). Third-generation sequencing, such as single-molecule real-time (SMRT) sequencing and Nanopore sequencing, is promising for DNA modification profiling, although the high error rate and poor quantification accuracy of third-generation sequencing need to be improved (Plongthongkum et al., 2014; Rhoads and Au, 2015; Rand et al., 2017; Simpson et al., 2017).

### Protein binding profiles of novel modifications and epigenetic manipulation

With the identification and characterisation of potential DNA modifiers and binding proteins, mechanisms for the remodeling and maintenance of novel modifications can be further investigated and thereby provide clues about their regulatory role and biological significance during early development. Modulation of expression of DNA modification-binding proteins are currently used for altering certain DNA modifications *in vivo*. However, these strategies cannot provide region-specific or subtle epigenetic changes which can be ‘matched’ with observed phenotype(s). With a comprehensive understanding of DNA modification-interacting proteins and CRISPR-related techniques, novel tools that allow epigenetic manipulation at a specific genomic region will provide direct evidence for correlations or causality between distinct epigenetic modifications and specific biological events, such as transgenerational epigenetic inheritance of disease risks (Magnani, 2014). Manipulation tools for several well-studied epigenetic modifications have already been applied (Liu X. S. et al., 2016; Thakore et al., 2016).

### “Rediscover” DNA modifications and the relationship with RNA modifications

The discovery of 6mA in eukaryotic genomes makes it tempting to speculate that the existence of DNA modifications in eukaryotic genomes is much more diverse than previously thought. Some DNA modifications detected at levels comparable to 5fC and 5caC were considered as well-established DNA lesions and therefore rarely investigated for biological significance. Recent studies suggest, other than causing genetic mutations, some of the rare DNA modifications may have biological functions via BER pathways, thereby representing a blur between DNA damage and epigenetic markers (Robertson et al., 2014; Ding et al., 2017; Fleming and Burrows, 2017).

On the other hand, modifications previously only in prokaryotic genomes or on RNA may be potential candidates for hitherto overlooked modifications in mammalian genomes. Some modifications and their binding proteins are ‘shared’ between DNA and RNA (Hudson and Ortlund, 2014). Similar to 6mA and its suggested modifier FTO, 5hmC, 5fC, and 5caC modification are also detected on RNA in mammals. Indeed, the latter mentioned RNA modifications have been suggested to be mediated by TET1 (Zhang H. Y. et al., 2016; Basanta-Sanchez et al., 2017). It would be of interest to identify the differences between DNA/RNA modifiers within a family that have significant preference towards DNA or RNA for further understanding of their functions and evolution. It is possible that during evolution these modifiers have shifted specificity from one type of nucleic acid to another, or lost their ‘dual’ specificity. Their ancient activities may not exist *in vivo* but might be observed in *in vitro* assays (Forterre and Grosjean, 2013).

RNA modifications may also be of great interest as they regulate RNA metabolism and gene expression at the translational level. RNA modifications and small non-coding RNAs are potential mediators of epigenetic inheritance in mammals. Recent reports indicate that malnutrition (Chen et al., 2016a; Sharma et al., 2016), stress/trauma (Gapp et al., 2014a; Rodgers et al., 2015) or exposure to toxins (Schuster et al., 2016) can lead to epigenetic inheritance mediated via small non-coding RNA in the male germline (Chen et al., 2016a; Watson, 2016). Elevated levels of 5-methylcytosine of RNA (m5C), which has been linked with increased stability of tRNA (Schaefer et al., 2010; Tuorto et al., 2012; Kiani et al., 2013), and N2-methylguanosine of RNA (m2G) have both been observed on small RNAs in sperm from mice fed high fat diets (HFD); this apparently is linked with the transmission of metabolic disorders to offspring (Chen et al., 2016a). That is, RNA modifications could contribute to the transmission of metabolic disorder via sperm small RNAs (Chen et al., 2016,b).

### Interplay and cooperation within the epigenome

The relative instability of RNA makes it unlikely to mediate epigenetic inheritance on its own. The interplay and cooperation among different epigenetic markers, which are still largely unclear, are likely to be critical for modulating biological events such as epigenetic inheritance and early embryonic development as well as for maintaining genome stability and cell identity during epigenetic reprogramming. A recent study of vinclozolin-induced transgenerational epigenetic inheritance indicates such association between differential methylated regions (DMRs) in F3 male germline, as early as E13, with sperm-borne small non-coding RNA (sncRNA) expression (Schuster et al., 2016). Co-existence of epigenetic modifications often indicate such cooperation within the epigenome and may reveal potential functions of novel DNA modifications. As mentioned above, apart from the correlation between 5mC and multiple histone modifications (Cedar and Bergman, 2009; Rose and Klose, 2014), 5hmC, 5fC, 5caC, and 6mA have also been reported to co-exist with certain histone modifications. New methods will be needed to detect multiple epigenetic modifications in the same context at individual loci within individual cells.

With more epigenetic modifications being discovered which can be highly dynamic, it will be necessary to establish databases curated for such information. Strides towards such goals are already in process. Recently, a manually curated database for epigenetic modifications during gametogenesis in seven mammals has been reported (Bai et al., 2017). Linkage between epigenetic modification databases with other databases such as interaction databases, pathway databases and disease-related databases would also be useful.

In summary, discovery and characterisation of novel DNA modifications are fascinating and fast-moving fields. DNA modifications often display extraordinarily dynamic patterns during early embryonic and germline development, yet the cause and consequence of these phenomena remain unclear. Therefore, exploring the dynamics of novel DNA modification during early development is a necessity for further understanding of DNA modifications in different biological contexts and a comprehensive epigenome map of mammalian genome.

## Author contributions

QZ, RS, and RA conceived the ideas and wrote the review.

### Conflict of interest statement

The authors declare that the research was conducted in the absence of any commercial or financial relationships that could be construed as a potential conflict of interest.
